# Process Evaluation of an Operational-Level Job Stress Intervention Aimed at Decreasing Sickness Absence among Public Sector Employees in Sweden

**DOI:** 10.3390/ijerph18041778

**Published:** 2021-02-12

**Authors:** Jonathan Severin, Lisa Björk, Linda Corin, Ingibjörg H. Jonsdottir, Magnus Akerstrom

**Affiliations:** 1 Region Västra Götaland, The Institute of Stress Medicine, 413 19 Gothenburg, Sweden; jonathan.severin@vgregion.se (J.S.); lisa.m.bjork@vgregion.se (L.B.); linda.corin@vgregion.se (L.C.); ingibjorg.jonsdottir@vgregion.se (I.H.J.); 2 Department of Sociology and Work Science, University of Gothenburg, 405 30 Gothenburg, Sweden; 3 Social Medicine, School of Public Health and Community Medicine, Institute of Medicine, The Sahlgrenska Academy at University of Gothenburg, 405 30 Gothenburg, Sweden; 4 Occupational Medicine, School of Public Health and Community Medicine, Institute of Medicine, The Sahlgrenska Academy at University of Gothenburg, 405 30 Gothenburg, Sweden

**Keywords:** occupational health, public sector, workplace intervention, process evaluation, organizational, sickness absence

## Abstract

Work-related sickness absence carries large societal costs, and interventions aimed at decreasing sickness absence need to be performed in an effective way. This study evaluated the implementation process of an operational-level job stress intervention, implemented between 2017 and 2018 in the public sector, by assessing the extent to which the allocated resources reached the intended target group, if the planned measures could be expected to address the relevant work environmental challenges, and if the planned measures were implemented. Data were collected from applications for funding in the intervention (*n* = 154), structured interviews (*n* = 20), and register data on sickness absence (*n* = 2912) and working conditions (*n* = 1477). Thematic analysis was used to classify the level of the work environmental challenges, the level and perspective of the suggested measures, and the “measure-to-challenge correspondence”. Overall, participating workplaces (*n* = 71) had both higher sickness absence (*p* = 0.01) and worse reported working conditions compared to their corresponding reference groups. A measure-to-challenge correspondence was seen in 42% of the measures, and individual-level measures were mostly suggested for organisational-level work environment challenges. Almost all planned measures (94%) were ultimately implemented. When performing operational-level interventions, managers and their human resource partners need support in designing measures that address the work environmental challenges at their workplace.

## 1. Introduction

Sickness absence is a concern that leads to substantial costs at all levels: for the individual, for their employer, and for society. For employers, costs include both the direct cost of absenteeism and the indirect costs generated from employee turnover and reduced productivity [1]. In 2018, the annual total costs to Swedish society due to sickness absence were estimated at 64 billion SEK (€6.2 billion) [2]. High sickness absence is common within the public sector in Sweden, especially in the health and social care sectors, with mental disorders such as depression, anxiety, and adjustment disorders being the most frequent diagnoses [3]. These mental disorders, and other stress-related mental health problems, including burnout, are associated with poor psychosocial working conditions such as high job demands, low job control, and low social support [4,5,6]. Intrapersonal relationships in work have also been shown to affect job satisfaction [7,8]. Hence, the workplace is an important arena both for reducing stress-related mental health problems, including burnout and sickness absence, and for promoting employees’ health [6,9,10,11,12,13,14].

In Sweden, employers are obliged to integrate a systematic work environment management system into their day-to-day activities, meaning that the employer should investigate occupational risks, implement preventive measures, and follow up these measures at the workplace to prevent ill-health and accidents, with the aim of achieving a sustainable work environment [15,16]. While top levels of management carry the overall responsibility for occupational health and safety at the workplace, line managers at lower organisational levels play a key role in turning this responsibility into action. The line managers’ systematic work managing the work environment is normally performed with the support of human resources (HR) partners and other support functions within the organisation. To succeed at this important task, knowledge about work environment and health is as crucial as skills in implementation and change management. It is also important that those performing these functions are aware of what is happening in the day-to-day activities at the workplace, and that they have the time and energy to systematically assess the workload and wellbeing among the employees [17,18]. However, if there is a high rate of sickness absences and/or extensive challenges within the working conditions at the workplace, temporary measures beyond the normal systematic work environment management, such as workplace interventions, might be needed to initiate a larger change in behaviours, routines, or practices to prevent occupational ill-health.

Workplace interventions can be performed with measures on different levels (individual, group, or organisational levels) and with different perspectives (promotive, preventive, or rehabilitative perspectives) [19,20]. Measures on an individual level aim to improve the physical or mental health of individuals, and often involve lifestyle activities [10,20]. Group-level measures often target the social interaction between individuals at the workplace [10,21,22]. Finally, measures on an organisational level target what could be referred to as the “causes of the causes”, aiming to ensure that structures, policies, and routines are designed to benefit occupational health [10,23]. When it comes to different perspectives, promotive measures refer to actions with a salutogenic approach, targeting empowerment and “the process of enabling people to increase control over, and to improve, their health” ([24,25] p. 422). Preventive measures assume a pathogenic perspective, aiming to eliminate or reduce risk factors and prevent ill-health [10,26,27]. Finally, a rehabilitative measure intends to cure illness or recreate health from a state of ill-health [19]. An intervention can encompass measures on one or more levels and be conducted from one or more perspectives, depending on its scope and aim [23,28,29].

When coming to terms with adverse working conditions, organisational-level interventions have been suggested to improve the “cause of the cause” [23,30,31,32]. Measures on an organisational level are also emphasised in the Swedish work environment regulations [16]. Ways of assessing the success of an intervention include examining its ability to be cost-effective [33] or effective in relation to its goal [34]. Systematic reviews have concluded that knowledge of the cost-effectiveness of organisational-level interventions is scarce [35,36]. Furthermore, evaluation of the effects of occupational health and safety interventions on an organisational level have shown varying results [37,38,39,40], partly because of the methodological challenges in evaluating such interventions [23,41,42]. Hence, more knowledge on the effectiveness of different approaches is needed in order to find optimal designs and implementation processes for organisational-level job stress interventions aimed at decreasing sickness absence among employees.

Process evaluations have been recommended as one way to gain such knowledge [23]. These evaluations can be used to “assess fidelity [i.e., adherence to the intention of the intervention] and quality of implementation, clarify causal mechanisms, and identify contextual factors associated with variation in outcomes” ([43] p. 3.) Furthermore, process evaluations can be used to understand and evaluate how interventions interact with the often-complex environment in which they are implemented, as there may be unpredictable or even undesired consequences [44]. They can also be used to understand why an intervention fails to produce the anticipated effects, whether the effects of an intervention are limited due to implementation failure, or because the causal chain for the intervention is weak [45]. To detect any weakness from assumptions regarding the causal chain, a logic model can be used to clarify these assumptions [46].

When performing a process evaluation, the evaluated aspects can be summarised in three different themes [44]: contextual factors, mechanisms of impact, and implementation. Contextual factors include “anything external to the intervention that may act as a barrier or facilitator to its implementation, or its effects” ([44] p. 2). Mechanisms of impact are used to clarify assumptions and to test and understand the pathways and mechanisms of impact. Finally, implementation includes the fidelity (quality of delivery), dose (quantity of delivery), and reach (to intended target groups) of the intervention [23]. An analysis of these themes can be used to understand why an intervention worked, or why it did not, which is important when interpreting intervention results [44,47,48].

In 2017, a special fund was allocated to decrease employee sickness absence and improve the work environment in a large organisation in Sweden. Operational-level line managers and their HR partners were invited to apply for financial resources from the fund to implement measures, beyond the legislated systematic work of managing the work environment, that could decrease sickness absence at their workplaces. The present study evaluated the implementation process of this large-scale job-stress intervention by assessing three aspects: firstly, the extent to which the allocated resources actually reached workplaces with high sickness absence and work environment challenges; secondly, whether the planned measures could be expected to address the identified work environmental challenges (i.e., the “measure-to-challenge correspondence”); and thirdly, whether the implemented measures differed from the non-implemented measures regarding the level (individual, group, or organisation), the perspective (promotion, prevention, or rehabilitation), or which stakeholders (HR, line managers, and/or occupational health services) were involved in the process of suggesting the measure and applying for funds.

## 2. Materials and Methods

### 2.1. Setting and Background

Sweden is divided into 21 regions, each with a regional council elected by the inhabitants. Each region is responsible for the public healthcare, transportation, culture, and development within its geographical area [49]. The region targeted for this intervention, Region Västra Götaland, is the largest employer within the public sector in Sweden, with approximately 55,000 employees. Around 85% of these employees work within the healthcare sector. From 2013 to 2019, the total sickness absence for the region’s employees varied between 5.5% and 6.8%. However, as in all large organisations, sickness absence also varies between departments and workplaces, where the total sickness absence ranged from 1% to 65% for different workplaces within the region in 2016. About 20% of the approximately 2800 workplaces (a group of employees and their line manager) within the region had a sickness absence exceeding 10% in 2016, measured before the intervention.

### 2.2. The Intervention

**Figure 1:**
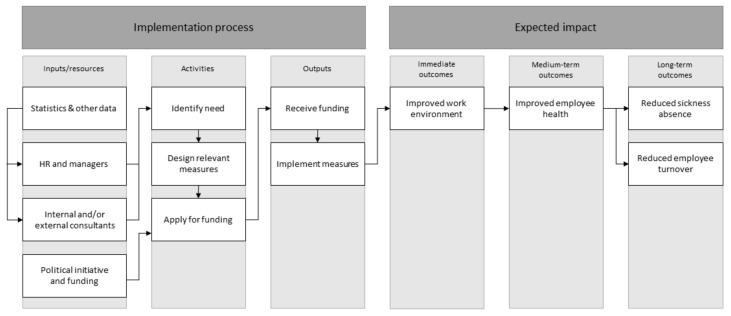
Schematic overview of the logic model of the intervention, focusing on the implementation process and expected impact.

Since 2017, an annual fund of approximately 1.5 million euros has been allocated by the regional council to decrease sickness absence and improve the work environment throughout the region. Line managers and their HR partners were invited to apply for financial resources from the fund to implement measures, beyond their legislated work of managing the work environment that could decrease sickness absence at their workplaces. The application guidelines stated that these measures should target conditions at the workplace level (i.e., organisational-level measures), rather than strengthen individual employees (i.e., individual-level measures) [28,50]. Managers were also given the opportunity to contact the internal occupational health service, free of charge, to obtain support when designing the measures. The applications were evaluated by a group of occupational health and safety experts, who decided whether or not to grant the funds. If funds were granted, the line managers were responsible for implementation, with or without support from the occupational health care services or other external experts. The present study concerns the process of the years 2017 and 2018, focusing primarily on evaluating the mechanism of impact and the implementation, since the contextual factors did not differ substantially between the intervention groups; they were all from the same sector and organisation, and participated in the intervention during the same timeframe. Furthermore, this study focuses on evaluating the implementation process of the intervention, where the evaluation of the impact, such as effects on work environment, sickness absence and employee turnover, is evaluated in a separate article [51]. An overview of the implementation process, together with causal assumptions for the expected impact, can be found in Figure 1.

### 2.3. Data Sources and Data Collection

**Figure 2:**
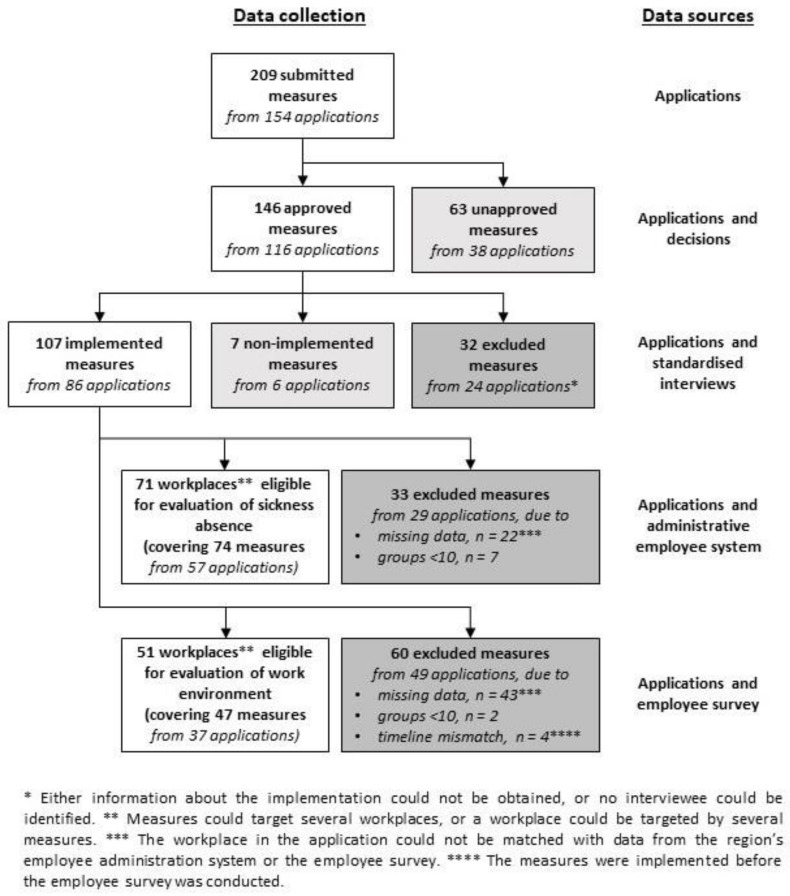
Flowchart describing the different steps in the data collection and the data sources used for each step, together with the number of measures, applications, and workplaces in each step.

For this process evaluation, several different data sources were used, and data were collected throughout the intervention process from four sources: (1) the applications and their corresponding decisions, (2) standardised phone interviews with HR partners who had knowledge of the granted measures, (3) the employee administration system, and (4) the employee survey. An overview of the different steps in the data collection and the data sources used is given in Figure 2 and will be explained in more detail below.

The applications were used to retrieve information on the type of organisation and type of workplace that applied for funding, as well as the targeted groups for the suggested measures (Table 1). Information on the work environment challenges and suggested measures described in the applications was used for content analysis.

Standardised interviews were conducted with HR partners in late 2019, by phone and/or email. The respondents were asked (1) whether the approved measures had been implemented, (2) when the measures were implemented, (3) if any changes had been made to the measures between being granted and being implemented, and (4) what the role of the person initiating the application was: line manager, HR partner, external consultant (such as a management consulting group), or the occupational health services. In total, 20 interviews were conducted, covering a mean of 5.7 measures per interview (ranging from 1 to 21 measures). One respondent could represent more than one workplace, and so these interviews provided information regarding 107 implemented measures and seven non-implemented measures.

Monthly data on total and short-term sickness absence (≤14 days) were collected from the employee administration system up to 12 months prior to the implementation of each intervention group’s approved measures. The sickness absence data were aggregated by intervention group (i.e., workplace) as well as by the operational area and department of which the intervention group was a part (i.e., two higher levels within the organisation). The latter groups served as reference groups (intervention groups deducted). Workplaces with less than 10 individuals were excluded (*n* = 7), giving a total of 71 workplaces eligible for evaluation of sickness absence, with a mean of 42 employees per workplace (ranging from 10 to 180 employees with a total of 2912 employees). Sickness absence was calculated as the percentage absence based on the number of hours of absence due to sickness divided by the total number of hours the group was expected to work each month (with vacation, parental leave, and caring for sick children deducted).

Self-reports on work environment conditions were collected from the region’s employee survey that was carried out in an online survey in September 2017, prior to the intervention. The survey was based on the widely known job demands–resources model and has been described in detail elsewhere [51]. Seven items were selected from the survey, covering workload, work situation, stress, motivation, effectiveness, and possibilities for recovery and reflection (shown in Table 2). Workplaces with fewer than 10 respondents were excluded (*n* = 2), giving a total of 51 workplaces eligible for evaluation of sickness absence, with a mean of 29 responding employees per workplace (ranging from 10 to 119 employees and a total of 1477 completed surveys). The overall regional response rate was 73%, ranging between 65 and 91% for the different departments within the region.

### 2.4. Data Analysis

First, to assess the extent to which the allocated resources actually reached the target group—that is, workplaces with high sickness absence—data on implemented measures eligible for the effect evaluation were analysed (74 measures within 57 applications from 71 workplaces; Figure 2). For each intervention group, a corresponding reference group was constructed from the aggregated means of the operational area and the department of which the intervention group was a part, with the intervention group excluded. When assumptions for parametric tests were not fulfilled (e.g., for sickness absence and the employee survey), the Mann–Whitney U-test was used to compare the aggregated means for total and short-term sickness absence for the intervention groups and their respective reference groups for a mean of 12, 6, and 3 months prior to the intervention, as sickness absence may vary over time. Since the suggested measures were implemented at different timepoints, all groups had different starting points, but the intervals were the same. To compare differences between the three different timepoints within the intervention groups, the Wilcoxon signed-rank test was used to examine median differences in these paired data. For work environmental challenges, the self-reports on work environment were used (47 measures within 37 applications from 51 workplaces). The analytical procedure described above was repeated, with the operational area being the only reference, but since individual answers were available, differences between each intervention group and its corresponding reference group could also be investigated.

Second, to evaluate whether the planned measures could be expected to address the described work environmental challenges, each application submitted to the intervention (209 measures from 154 applications; Figure 2) was analysed using a deductive theme analysis with predetermined categories [52]. The applications were classified according to the level of the challenge, the level of the suggested measure (individual, group, or organisational) and the perspective of the suggested measure (promotion, prevention, or rehabilitation). The challenges were also classified according to type of challenge (e.g., shortage of staff or high workload) and type of suggested measure (e.g., workshop and team building).

This second section of the analysis also included an investigation of the motivations for the suggested measures, including the so-called “measure-to-challenge correspondence”. The applications were assessed and dichotomised in terms of whether the measures suggested were clearly motivated according to the complexity of the challenges described (yes/no). Even if a challenge was judged to be organisational, there could still be motivation to suggest measures on other levels related to this organisational challenge. For example, one application described high sickness absence, high workload, high employee turnover, and situations related to threats and violence from patients, which were all classified as organisational-level challenges. The measure suggested was offering the employees education on how to deal with violence and threats from patients; although this was classified as an individual-level measure, it was judged as “yes” with regard to measure-to-challenge correspondence. All classifications were performed independently by two researchers (with expertise in public health science and organisational work environment, respectively), and congruence was assessed. In total, 15% of the classifications differed: 10/209 for the level of described challenges, 42/209 for the level of suggested measures, 35/209 for the perspective of suggested measures, and 36/209 for the measure-to-challenge correspondence. Differences in classifications were discussed until consensus was reached.

Third, to evaluate whether there were any differences between implemented and non-implemented measures in terms of descriptive data from the applications, the classifications of applications as well as interview data were used. Information on implementation and stakeholder involvement was retrieved from the interviews. Chi-squared tests were used to analyse differences in categorical data between groups.

Version 25 of IBM SPSS Statistics (IBM, Armonk, New York, NY, USA) was used for all statistical analysis. Statistical significance was set at *p* < 0.05, and two-sided confidence intervals were used.

## 3. Results

The numbers of measures, applications, and workplaces in the different steps of this process evaluation (submitted measures, granted measures, implemented measures, and measures eligible for an effect evaluation) are given in Figure 2. The types of organisation and workplace behind the submitted applications, together with information on the targeted groups and roles that initiated the applications, can be found in Table 1.

### 3.1. Comparison of Sickness Absence before the Intervention between the Intended Target Groups and Their Reference Groups

Overall, both total sickness absence and short-term sickness absence were significantly higher in the intervention groups than in their respective reference groups (i.e., operational area and department) at 12, 6, and 3 months prior to implementation of the measures (2017 and 2018) (Table 3). When comparing sickness absence at different times within the intervention groups, total sickness absence was higher 3 months before the intervention started compared to 12 months before (*p* < 0.001), but there was no difference between 3 and 6 months (*p* = 0.05), while short-term sickness absence was significantly higher 3 months before the intervention started than 6 and 12 months before (*p* < 0.001).

### 3.2. Comparison of Work Environment before the Intervention between the Intended Target Groups and Their Reference Groups

Significant differences in the work environment between the intervention and reference groups, with poorer results for the intervention groups, were seen for two items in the employee survey: looking forward to work and time for reflection (Table 2). The structure of the data also enabled comparison between each intervention group and its corresponding reference group for each item (357 comparisons in total). In 44 of these comparisons, significantly poorer working conditions were noted for the intervention group compared to the reference group whereas, in 33 comparisons, poorer working conditions were noted for the reference group compared to the intervention group.

### 3.3. Description of the Work Environmental Challenges in the Applications

Almost all applications (*n* = 203, 97%), contained descriptions of organisational-level work environmental challenges (Table 4). Four main types of challenge could be observed: (1) shortage of staff due to a high employee turnover or vacant positions due to difficulties in recruiting staff with the right competence, (2) increased workload, for example, due to an inflow of new patient groups or downsizing of the operations, (3) ethical stress due to caring for specifically vulnerable patient groups or being forced to prioritise between different tasks affecting the patients, and (4) unclear goals, tasks, or other challenges due to reorganisation of the operations. Work environment challenges described on a group level (*n* = 6, 3%), included lack of social support, trust, communication, and/or cooperation, often resulting in a poor working environment with conflicts or victimisation between groups of employees or between employees and managers. No individual-level challenge was observed.

### 3.4. Description of Suggested Measures in the Applications

Almost all of the 209 suggested measures had a preventive perspective (*n* = 183, 88%), whereas the remaining ones had a promotive (*n* = 18, 9%) or a rehabilitative (*n* = 8, 4%) perspective. The distribution of measures between the individual, group, and organisational levels was *n* = 87 (42%), *n* = 55 (26%), and *n* = 67 (32%), respectively (Table 4). No differences in the level (*p* = 0.2) or perspective of the measure (*p* = 0.5) were found between granted and non-granted measures. With regard to the type of measure, common types of measure suggested on an individual level were lectures and workshops, often aiming to inspire, motivate, and/or support individual employees in improving their lifestyle (e.g., diet and physical activity) or providing them with personal strategies to manage their situation. Another type was physical activity; for example, employees were given an opportunity to get coaching or scheduled physical activity during the work shift to improve their level of physical activity. Other individual-level measures included massage, wellness activities, and medical examinations. The main type of group-level measure was teambuilding; for example, to improve the group dynamics or to strengthen the cooperation between employees at the workplace. Types of organisational-level measures included work environmental analyses (e.g., assessments to identify challenges or developing action plans on how to improve the work environment), manager support, and structural changes including schedule improvements or improvements involving day-to-day routines within the operation.

In terms of comparisons between the level of the described challenge and the level of the suggested measure, all of the applications describing group-level challenges suggested group-level measures (*n* = 6, 3%). For challenges described on an organisational level (*n* = 203, 97%), approximately one third of the applications suggested measures at the same level (*n* = 67, 32%), but as many as *n* = 87 (42%) suggested individual measures and *n* = 49 (23%) suggested group measures (Table 4).

### 3.5. Motivation for Selecting Different Measures and Measure-To-Challenge Correspondence

**Figure 3:**
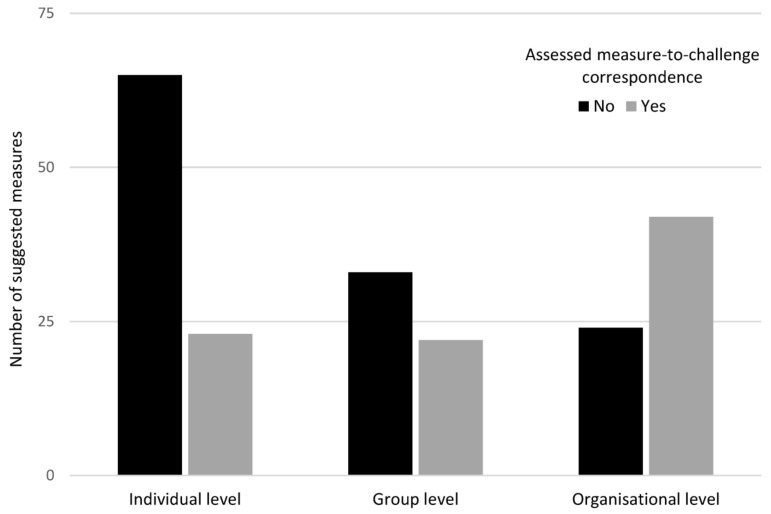
Number of suggested measures (*n* = 209 in total) divided by the level of the measures and stratified by measure-to-challenge correspondence (yes/no).

When analysing the motivation for the suggested measures, three main types of motivations emerged: (1) the measure had been shown to have promotional or preventive effects, (2) the measure needed to be executed in order to identify the “cause of the cause” or to identify how to affect the “cause of the cause”, and (3) the need for this measure had been shown in the systematic work of managing the work environment. An analysis was also conducted to assess the “measure-to-challenge correspondence”; that is, whether the applications contained a clear motivation for the suggested measures. This was the case for *n* = 87 (42%) of the total suggested measures (*n* = 209). Organisational level measures were suggested more often when this correspondence was present (48% of all suggested measures with this correspondence compared to 36% of all suggested measures with no correspondence, *p* < 0.001), as shown in Figure 3. No differences in measure-to-challenge correspondence were seen with regard to whether HR (*p* = 0.06), the internal occupational health service (*p* = 1), or any other external expert resources (*p* = 1) participated in the development of the measures.

### 3.6. Did Implemented Measures Differ from Non-Implemented Measures?

After exclusion of measures that could not be followed up, *n* = 107 (94%) of all granted measures were implemented as planned or with minor adjustments (Figure 2). There were no differences between the implemented and non-implemented measures in terms of measure level (*p* = 0.2), perspective (*p* = 0.6), measure-to-challenge correspondence (*p* = 0.2), or whether an internal consultant (*p* = 0.8), external consultant (*p* = 0.7), or the occupational health services (*p* = 0.3) were involved in suggesting the measure.

## 4. Discussion

This study assessed the implementation process of a large-scale job stress intervention performed at the operational level in the Swedish public sector. Investigating whether the allocated resources reached the intended target group, whether the planned measures could be expected to address the work environmental challenges of the intervention groups, and whether planned measures were implemented offers the possibility of finding plausible explanations for why an organisational intervention might have succeeded or failed. Thus, this study can assist future endeavours to decrease employee sickness absence by providing insights on intervention design and implementation.

### 4.1. Did the Intervention Reach the Target Group?

The results showed that the intervention reached the intended target group; that is, workplaces with high sickness absence and/or adverse working conditions. In comparison to the reference groups, participating workplaces had an overall higher total and short-term sickness absence, and, to some extent, poorer working conditions. The differences between the intervention and reference groups were consistent when analysing sickness absence 3, 6, and 12 months before the intervention, showing that these workplaces had experienced challenges for at least a year prior to the intervention. Taken together, these results indicate a successful initiation phase of the intervention.

### 4.2. Did the Suggested Measures Correspond to the Work Environmental Challenges?

Less than half of all the suggested measures were found to correspond to the work environmental challenges described in the corresponding applications. Work environmental challenges on an organisational level were, to a large extent, addressed by individual-level measures, a tendency that has been observed in other studies [28,38,50,53]. The present study introduces the concept of “measure-to-challenge correspondence” to describe the agreement between an existing problem and the suggested solution. Thus, measures targeting levels other than the one that was primarily identified as the source of the challenge could be clearly motivated as a part of the solution, even though this measure might not solve “the cause of the cause” on an organisational level.

Due to their key position in the organisation, line managers often possess invaluable knowledge about the workplace—knowledge which is necessary for achieving a good “fit” between intervention and context [23]. They also need extensive knowledge about psychosocial work environment management [54,55], interpersonal relationship [8] and change management [56,57]. The low measure-to-challenge correspondence found in this study indicates that both line managers and their HR partners need support when it comes to these issues. The fact that managers need to be supported by their organisations with adequate structures, routines, and a sound culture for occupational health and safety is underscored both in the Swedish legislation and in the literature [18,58]. This low measure-to-challenge correspondence and the overall fact that individual measures were most commonly suggested in applications with clear organisational challenges is plausibly an important factor in explaining the outcome of the intervention.

In the current study, line managers had access to both additional funding and support from their own organisation, that is, from expert functions within HR or the occupational health services, which they used to a high extent. However, the involvement of HR, the internal occupational health services, or other external expert resources did not influence the measure-to-challenge correspondence, showing a need for further support in designing measures that better address the work environmental challenges at hand. This could be done by providing the occupational health services and HR with efficient tools for supporting line managers in designing measures within the intervention. Another way of improving the measure-to-challenge correspondence would be to add a new criterion for whether an application is granted, covering whether the planned measure could be expected to address the work environmental challenges at hand. Increasing this correspondence is especially important, since a high measure-to-challenge correspondence has been identified as an important factor in decreasing sickness absence [31,50,59,60,61].

### 4.3. Were the Planned Measures Implemented?

Since the quantity of delivery within an intervention could also help explain why an intervention worked or did not work [23], this study also assessed whether the measures were implemented as planned. Overall, most measures were implemented as planned, or with minor adjustments, which might be due to the active involvement of the line managers [23,62,63,64]. It should be noted that there was a substantial loss of data during this step of the process evaluation; the follow-up interviews were performed between 1 and 2 years after the intervention, resulting in the loss of key personnel with knowledge of the participation in the intervention due to employee turnover or organisational changes. However, a comparison between the measures that could be verified as implemented and the measures that were not implemented did not reveal any systematic differences. This indicates that the loss of data did not introduce a systematic bias in this process evaluation.

### 4.4. Strengths and Limitations

Performing a process evaluation of an operational-level job-stress intervention in a real-life setting will result in both strengths and limitations. Firstly, using register data from the employee on sickness absence instead of self-assessed sickness absence offers higher data quality. However, in our study it also resulted in a large loss of data due to incongruence between different administrative systems. The workplaces with missing data did not differ from the rest of the workplaces, and so this could potentially lead to an underestimation of the true intervention effect rather than affecting the overall result of this study. Secondly, it was not possible to assess the extent or contexts of non-participating workplaces with high sickness absence and poor working conditions, which might lead to an uncertainty as to whether the intended target group was reached or not. Comparison to an earlier evaluation showing that 20% of 2800 workplaces exceeded, in 2017, a sickness absence of 10%, may indicate that there are many workplaces in the intended target group that did not apply for intervention funding. However, a high sickness absence does not necessarily imply a poor work environment, since a high sickness absence within a workplace might be caused by clusters of long-term sickness absence from illnesses unrelated to the working conditions. Data on working conditions were restricted to single items used in the regions’ employee survey and potential effects not included among these items could not be investigated. We were also limited to surveys distributed in September 2017. Thus, the pre-measurements were not adjusted to the time frame for the individual measures. Another important limitation is the possibility of a lack of objectivity and impartiality from the HR partners participating in the interviews, mainly regarding the judgement as to the measures had been implemented or not. Finally, it was not possible to verify exactly what was done in each implementation process, and so the information on the implemented measures was drawn from the initial applications to the intervention. Adjustments could have been made during the implementation by the workplaces themselves, by the occupational health services, or by the external experts involved in the implementation, which could have improved the measure-to-challenge correspondence. Information on changes to the measures during implementation was requested during the phone interviews, but this information was hard to retrieve. The measure-to-challenge correspondence could also be potentially higher due to measures implemented outside the intervention. However, reading the submitted applications did not give any indications of managers implementing further measures to address the same challenges.

## 5. Conclusions

In order to increase the efficiency of organisational-level job-stress interventions, the processes of their design and implementation must be improved. This study investigated the implementation of a large-scale job-stress intervention in terms of whether the intervention reached the intended target group, the extent to which the planned measures could be expected to address the work environmental challenges, and whether these measures were actually implemented. The findings suggest that the intervention reached the targeted population; that is, workplaces with high levels of work environmental challenges and high sickness absence. They also suggest a high degree of implementation of the measures suggested to address these issues. However, the main finding of this study is that the actors who suggested the measures—line managers and their HR partners—struggled to match the work environmental problems with adequate solutions, resulting in a low measure-to-challenge correspondence. This lack of correspondence will probably influence the extent to which the intervention succeeds in improving working conditions and reducing sickness absence. Thus, the overall results show that financial resources are not enough for successful interventions. To tackle the complexity of work environment and job stress, managers and HR need support in designing measures with a good fit to the context.

## Figures and Tables

**Table 1:** Descriptive background data for the workplaces behind the 154 applications submitted to the intervention.

	Applications, *n* (%)
Type of organization	
- Major hospital	68 (44)
- Minor hospital	56 (36)
- Primary care	15 (10)
- Service and maintenance	12 (8)
- Culture	3 (2)
Type of workplace	
- Health care	106 (69)
- Administration	28 (18)
- Service	12 (8)
- Management	8 (5)
Targeted group in the application	
- All employees	114 (74)
- Subgroup of employees	30 (20)
- Managers only	10 (6)
Role that initiated the application	
- Line manager and HR together	97 (63)
- Line manager without HR	31 (20)
- HR without the line manager	26 (17)

**Table 2:** Mean group scores for evaluated items in the employee survey (carried out in September 2017) prior to the intervention for the intervention and reference groups, and *p*-values for the differences between these groups.

Variable	Item Wording	Response Scale (Min–Max)	Intervention Groups * (*n* = 51)		Reference Groups * (Operational Areas) (*n* = 51)		
			Mean	Mean Range (Min–Max)	Mean	Mean Range (Min–Max)	*p*-Value (Difference in Mean)
Recovery	“I have scope for recovery during the working day through breaks and/or rests.”	*1 = Strongly disagree–* *5 = Strongly agree*	3.22	1.91–4.09	3.27	1.91–4.06	0.96
Stress **	“Do you feel stressed currently? (Stress means a condition in which you feel tense, nervous, or uneasy, or are unable to sleep at night because you are thinking about problems the whole time).”	*1 = None–5 = Very much*	2.82	1.87–3.90	2.70	2.29–3.27	0.1
Quantity of work	“The quantity of my work seems reasonable.”	*1 = Strongly disagree–* *5 = Strongly agree*	3.13	1.72–4.18	3.19	1.94–4.11	0.9
Looking forward to work	“I look forward to going to work.”	*1 = Strongly disagree–* *5 = Strongly agree*	3.77	2.82–4.53	3.89	3.45–4.23	0.008
Reflection	“I have time for reflection and consideration in my work.”	*1 = Strongly disagree–* *5 = Strongly agree*	2.88	1.55–3.85	2.93	1.77–3.78	<0.001
Effectiveness	“How satisfied are you with the effectiveness of the work performed by your unit/department? (Effectiveness here means doing the right things in the right way in the right order and without any unnecessary waste of time).”	*1 = Very dissatisfied–* *5 = Very satisfied*	3.35	2.56–3.98	3.27	2.31–4.0	0.2
Work situation	“How satisfied are you with your current work situation?”	*1 = Very dissatisfied–* *5 = Very satisfied*	3.49	1.91–4.4	3.60	2.75–4.22	0.08

* mean survey score calculated as the mean of the individual group mean, ** reversed question where a low score is preferred.

**Table 3:** Average levels and range of total and short-term sickness absence registered 12, 6, and 3 months prior to implementation of the measures (2017 and 2018), in the intervention and reference groups, and *p*-values for the respective differences between these groups.

			Reference Groups			
	Intervention Groups (*n* = 71)		Operational Areas (*n* = 67) *		Departments (*n* = 71)	
	Mean	Mean Range (Min–Max)	Mean (*p*-Value between Intervention Groups and Operational Area)	Mean Range (Min–Max)	Mean (*p*-Value between Intervention Groups and Department)	Mean Range (Min–Max)
Total sickness absence						
12 months	8.3%	0.7–17.9%	7.0% (*p* = 0.008)	1.3–12.6%	7.1% (*p* = 0.001)	6.3–9.1%
6 months	9.1%	0.3–19.8%	7.6% (*p* = 0.007)	1.6–13.5%	7.7% (*p* = 0.001)	6.9–9.8%
3 months	9.3%	0.6–22.1%	7.8% (*p* = 0.012)	1.3–14.1%	8.0% (*p* = 0.001)	7.4–9.7%
Short-term sickness absence (≤14 days)						
12 months	2.9%	0.4–4.8%	2.5% (*p* = 0.003)	1.1–3.7%	2.6% (*p* < 0.001)	2.1–3.4%
6 months	3.5%	0.3–6.1%	3.0% (*p* = 0.001)	1.3–4.4%	3.1% (*p* < 0.001)	2.5–3.9%
3 months	3.9%	0.6–8.1%	3.3% (*p* = 0.005)	1.3–5.1%	3.4% (*p* < 0.001)	2.9–4.4%

* For four intervention groups, data on the level of operational area could not be collected due to the organisational structure; that is, the intervention group equalled the operational area, or constituted too large a part of the operational area.

**Table 4:** Levels of the challenges described and measures suggested for the 209 submitted measures.

		Level of Described Challenge			
		Individual	Group	Organisational	Sum
**Level of suggested measure**	Individual	0 (0%)	0 (0%)	87 (42%)	87 (42%)
	Group	0 (0%)	6 (3%)	49 (23%)	55 (26%)
	Organisational	0 (0%)	0 (0%)	67 (32%)	67 (32%)
	Sum	0 (0%)	6 (3%)	203 (97%)	209 (100%)

## Data Availability

Data available on request.
